# Brown adipose tissue plays a central role in systemic inflammation-induced sleep responses

**DOI:** 10.1371/journal.pone.0197409

**Published:** 2018-05-10

**Authors:** Éva Szentirmai, Levente Kapás

**Affiliations:** 1 Elson S. Floyd College of Medicine, Department of Biomedical Sciences, Washington State University, Spokane, Washington, United States of America; 2 Sleep and Performance Research Center, Washington State University, Spokane, Washington, United States of America; State University of Rio de Janeiro, BRAZIL

## Abstract

We previously identified brown adipose tissue (BAT) as a source of sleep-inducing signals. Pharmacological activation of BAT enhances sleep while sleep loss leads to increased BAT thermogenesis. Recovery sleep after sleep loss is diminished in mice that lack uncoupling protein 1 (UCP-1), and also in wild-type (WT) mice after sensory denervation of the BAT. Systemic inflammation greatly affects metabolism and the function of adipose tissue, and also induces characteristic sleep responses. We hypothesized that sleep responses to acute inflammation are mediated by BAT-derived signals. To test this, we determined the effects of systemic inflammation on sleep and body temperature in UCP-1 knockout (KO) and WT mice. Intraperitoneal injections of lipopolysaccharide, tumor necrosis factor-α, interleukin-1 beta and clodronate containing liposomes were used to induce systemic inflammation. In WT animals, non-rapid-eye movement sleep (NREMS) was elevated in all four inflammatory models. All NREMS responses were completely abolished in UCP-1 KO animals. Systemic inflammation elicited an initial hypothermia followed by fever in WT mice. The hypothermic phase, but not the fever, was abolished in UCP-1 KO mice. The only recognized function of UCP-1 is to promote thermogenesis in brown adipocytes. Present results indicate that the presence of UCP-1 is necessary for increased NREMS but does not contribute to the development of fever in systemic inflammation.

## Introduction

There are complex interactions among metabolism, inflammation and sleep. Inflammation and changes in metabolic status modulate sleep while sleep and sleep loss affect metabolism and the outcome of infections [1–4]. Inflammation brings about characteristic metabolic responses while pathological changes in metabolic status, such as in obesity, are often accompanied by systemic inflammation [5–7]. Shared central regulatory circuits and peripheral signaling and effector mechanisms are likely to underpin the tight connection among inflammation, metabolism and sleep.

Brown adipose tissue (BAT) plays a central role in regulating metabolism and generating metabolic signals that modulate sleep. It is a key effector organ in maintaining metabolic homeostasis by adjusting energy expenditure, glucose disposal and adaptive heat production [8]. The thermogenic property of BAT is conferred by the tissue-specific presence of uncoupling protein 1 (UCP-1) in the mitochondria of brown adipocytes [9]. Proinflammatory cytokines activate the thermogenic machinery in BAT [10–13] but the extent to which increased BAT thermogenesis contributes to fever in systemic inflammation is unclear. In recent studies we demonstrated that signals arising from activated BAT play an important role in sleep regulation, and pharmacological activation of BAT greatly enhances sleep [14,15]. Sleep deprivation induces increased BAT thermogenesis which, in turn, is permissive for subsequent recovery sleep [14]. The sleep-inducing signals from activated BAT are independent of changes in body temperature and likely reach the central nervous system by capsaicin-sensitive BAT afferents [14].

Systemic inflammations are accompanied by fever, loss of appetite, decreased activity, social withdrawal and increased sleep, collectively known as sickness syndrome [16,17]. Bacterial or viral infections, administration of bacterial cell wall components or viral double-stranded RNA enhance non-rapid-eye movement sleep (NREMS) (reviewed in [2]). Proinflammatory cytokines, such as tumor necrosis factor alpha-α (TNFα) and interleukin-1 beta (IL-1β) are likely key mediators of this response by acting on peripheral target sites [2]. Since proinflammatory cytokines activate BAT and activated BAT is a source of somnogenic signals, we hypothesized that BAT contributes to enhanced sleep during systemic inflammation.

To induce acute, systemic inflammation we used intraperitoneal (i.p.) injections of either TNFα, IL-1β, bacterial lipopolysaccharide (LPS) or clodronate containing liposomes (CCL). LPS, or endotoxin, is a heat-stable, biologically active component of the outer membrane of Gram-negative bacteria and a highly proinflammatory molecule. Peripheral administration of LPS induces a wide range of biological effects such as fever, anorexia, increased NREMS and decreased rapid-eye movement sleep (REMS) in rats and mice [18–22]. These effects of LPS are mainly due to the induction of TNFα, IL-1β and other proinflammatory mediators [23].

Peripheral administration of CCL has been widely used for macrophage depletion. Macrophages phagocytose and digest CCL; free clodronate accumulates intracellularly and induces the disintegration of the cells with the concomitant release of cytokines and other bioactive proinflammatory substances [24,25]. The acute effects of CCL administration are very similar to those of LPS and proinflammatory cytokines, which include increases in NREMS and body temperature, with a simultaneous decrease in REMS and electroencephalographic slow-wave activity (EEG SWA) [26].

To test the hypothesis that thermogenic activation of BAT is a mediator of sleep responses to systemic inflammation, we investigated the effects of proinflammatory stimuli on sleep and body temperature in UCP-1 deficient mice. We report here that sleep responses, but not fever, are completely abolished in UCP-1 deficient mice in four models of acute, systemic inflammation indicating that BAT thermogenesis is a key signal for increased sleep but does not contribute to the development of fever.

## Materials and methods

### Animals

All animal husbandry and experimental procedures were carried out in accordance with the Association for Assessment and Accreditation of Laboratory Animal Care (AAALAC) and approved by the Institutional Animal Care and Use Committee (IACUC) of the Washington State University (protocol number 4762). All animal work was conducted in accordance with the recommendations in the Guide for the Care and Use of Laboratory Animals of the National Institutes of Health. Male, 4–5 months old C57BL/6 and congenic UCP-1 knockout (KO) mice were used in the experiments. Breeding pairs of the UCP-1 KO animals were obtained from Dr. Leslie P. Kozak of Pennington Research Institute (Baton Rouge, LA). Breeding pairs of C57BL/6 mice were purchased from the Jackson Laboratory. Both genotypes were further bred at Washington State University under identical environmental conditions. Each mouse used in the experiments was genotyped (Transnetyx, Cordova, TN).

### Surgery

All surgical procedures were performed with the mice under ketamine-xylazine anesthesia (87 and 13 mg/kg, respectively) and all efforts were made to minimize suffering. For sleep-wake activity recordings, the animals were implanted with three cortical EEG electrodes, placed over the frontal and parietal cortices, and two nuchal electromyographic (EMG) electrodes. The EEG and EMG electrodes were anchored to the skull with dental cement. Telemetry transmitters were implanted intraperitoneally for body temperature and motor activity recordings. All mice were allowed to recover from surgery for at least 10 days before any experimental manipulation started and handled daily to adapt them to the experimental procedures. Mice were housed individually in sound-attenuated environmental chambers on a 12-h light/12-h dark cycle (lights on at 4 am) and 30 ± 1^°^C ambient temperature. Regular rodent chow (Harlan Teklad, Product No. 8460) and water were available *ad libitum* throughout the experiments. The body weight of the wild type (WT) and UCP-1 KO mice at the time of the experiment was 28.2 ± 0.6 g and 27.5 ± 0.6 g, respectively.

### Sleep-wake activity recordings and analyses

The mice were tethered to commutators, which were further routed to Grass Model 15 Neurodata amplifier system (Grass Instrument Division of Astro-Med, Inc., West Warwick, RI). The amplified EEG and EMG signals were digitized at 256 Hz and recorded by computer. The high-pass and low-pass filters for EEG signals were 0.5 and 30.0 Hz, respectively. The EMG signals were filtered with low and high cut-off frequencies at 100 and 10,000 Hz, respectively. The outputs from the 12A5 amplifiers were fed into an analog-to-digital converter and collected by computer using SleepWave software (Biosoft Studio, Hersey, PA). Sleep-wake states were scored visually off-line in 10-s segments. The vigilance states were defined as NREMS, REMS and wakefulness according to standard criteria as described previously [22]. Time spent in wakefulness, NREMS and REMS was calculated in 2-, 6- and 12-h blocks. EEG power data from each artifact free 10-s segment were subjected to off-line spectral analysis by fast Fourier transformation. EEG power data in the range of 0.5 to 4.0 Hz during NREMS were used to compute EEG SWA. EEG SWA data were normalized for each animal by using the average EEG SWA across 24 h on the baseline day as 100. The results were further averaged in 2-h bins.

### Telemetry recordings

Core body temperature and locomotor activity were recorded by MiniMitter telemetry system (Starr Life Sciences Corp.) using VitalView software. Temperature and activity values were collected every 1 and 10 min, respectively, throughout the experiment and were averaged over 2-h time blocks.

### Experimental procedures

**Experiment 1****:** The effects of TNFα on sleep-wake activity, body temperature and metabolic parameters in WT and UCP-1 KO mice.

WT and UCP-1 KO mice (n = 7 for both genotype) received i.p. injections of saline on the baseline day and TNFα (GenScript, Piscataway, NJ) on the test days 10–15 min before the onset of the dark period. The two doses of TNFα were of 0.3 and 1 μg/mouse. All the mice were injected with both doses of TNFα with 6 days between the TNFα injection days. Sleep-wake activity and body temperature were recorded for 24 h after saline and TNFα injections.

In a separate group of mice (n = 6 for WT and n = 8 for UCP-1 KO mice) oxygen consumption (VO_2_, ml/kg/h) and carbon dioxide production (VCO_2_, ml/kg/h) were measured with indirect calorimetry (Oxymax System, Columbus Instruments, Columbus, OH). Twenty-four-hour food intake (g/kg/h) was also recorded. All three measurements were taken every 10 min and the data were collapsed into 24-h bins. Respiratory exchange ratio (RER; VCO_2_/VO_2_) was calculated. The animals were habituated to the calorimetry cages for three days before the experiments. On the fourth day mice were injected with saline i.p. (baseline), on the fifth day they received 1 μg TNFα (test day).

**Experiment 2****:** The effects of IL-1β on sleep-wake activity and body temperature in WT and UCP-1 KO mice.

WT (n = 6) and UCP-1 KO mice (n = 7) received i.p. injections of saline on the baseline day and 0.4 μg/mouse IL-1β (GenScript, Piscataway, NJ) on the test day 10–15 min before the onset of the dark period. Sleep-wake activity and body temperature were recorded for 24 h after saline and IL-1β injections.

**Experiment 3****:** The effects of LPS on sleep-wake activity and body temperature in WT and UCP-1 KO mice.

WT and UCP-1 KO mice (n = 7 for both genotype) received i.p. injections of saline on the baseline day and 100 μg/kg LPS (Sigma-Aldrich, E. coli O111:B4) on the test day 10–15 min before the onset of the dark period. Sleep-wake activity and body temperature were recorded for 24 h after saline and LPS injections.

**Experiment 4****:** The effects of CCL on sleep-wake activity and body temperature in WT and UCP-1 KO mice.

On the baseline day (day 1), all mice (n = 7 for both WT and UCP-1 KO mice) were injected i.p. with saline. On the next day, all mice received CCL i.p. (1.2 mg/mouse in 0.2 ml volume, FormuMax Scientific, Inc, Palo Alto, CA). Injections were performed 5–10 min before the onset of the dark phase. Recordings started immediately after saline and CCL the injections for 24 h.

### Statistics

Time spent in wakefulness, NREMS and REMS, as well as, EEG SWA, body temperature and motor activity were calculated in 2, 6- and 12-hour blocks; 3-way mixed ANOVA was performed separately for each treatment across 24 h on 2-h data blocks (independent measure: genotype, repeated measures: time and treatment). When ANOVA indicated significant effects, Student-Newman-Keuls test was used as the *post hoc* test for all experiments. Daily food intake, VO_2_ and RER were analyzed by using two-way mixed ANOVA (independent measure: genotype, repeated measure: treatment). Changes in these parameters within the same animal groups were analyzed by paired t-test; differences between groups were analyzed by Student’s t-test. An α-level of P < 0.05 was considered to be significant.

## Results

### The effects of systemic inflammation on NREMS

**Fig 1:**
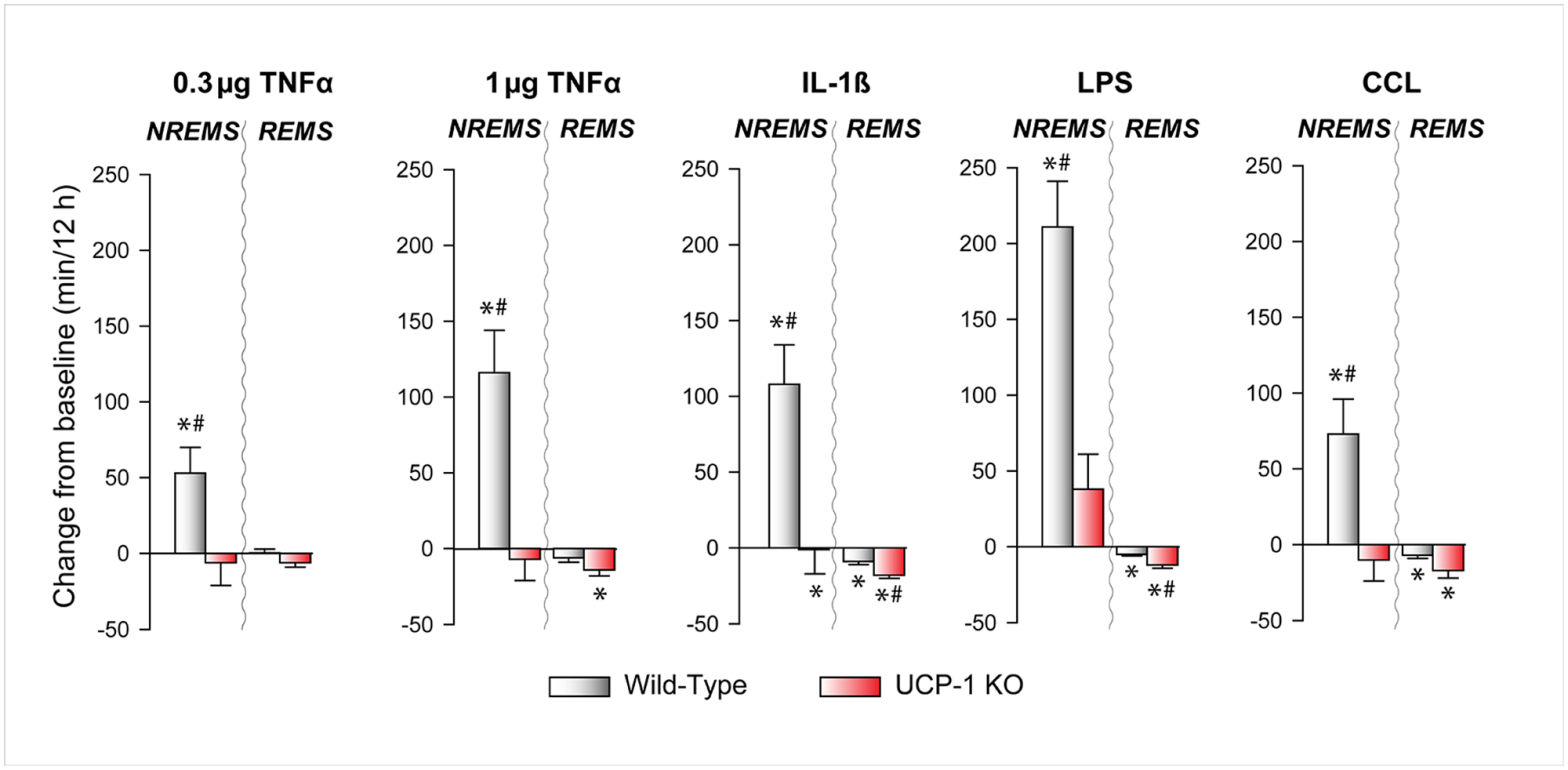
The effects of tumor necrosis factor-α (TNFα), interleukin 1β (IL-1β), lipopolysaccharide (LPS) and clodronate-containing liposomes (CCL) on non-rapid-eye movement sleep (NREMS) and rapid-eye movement sleep (REMS) in wild-type (WT) and uncoupling protein 1 knockout (UCP-1 KO) mice. Grey bars: WT mice; red bars: UCP-1 KO mice. Data are expressed as difference from baseline during the first 12 h after the treatments. * significant difference from baseline within the same genotype (paired t-test, p < 0.05); # significant difference between genotypes (Student’s t-test, p < 0.05); error bars: SE.

**Fig 2:**
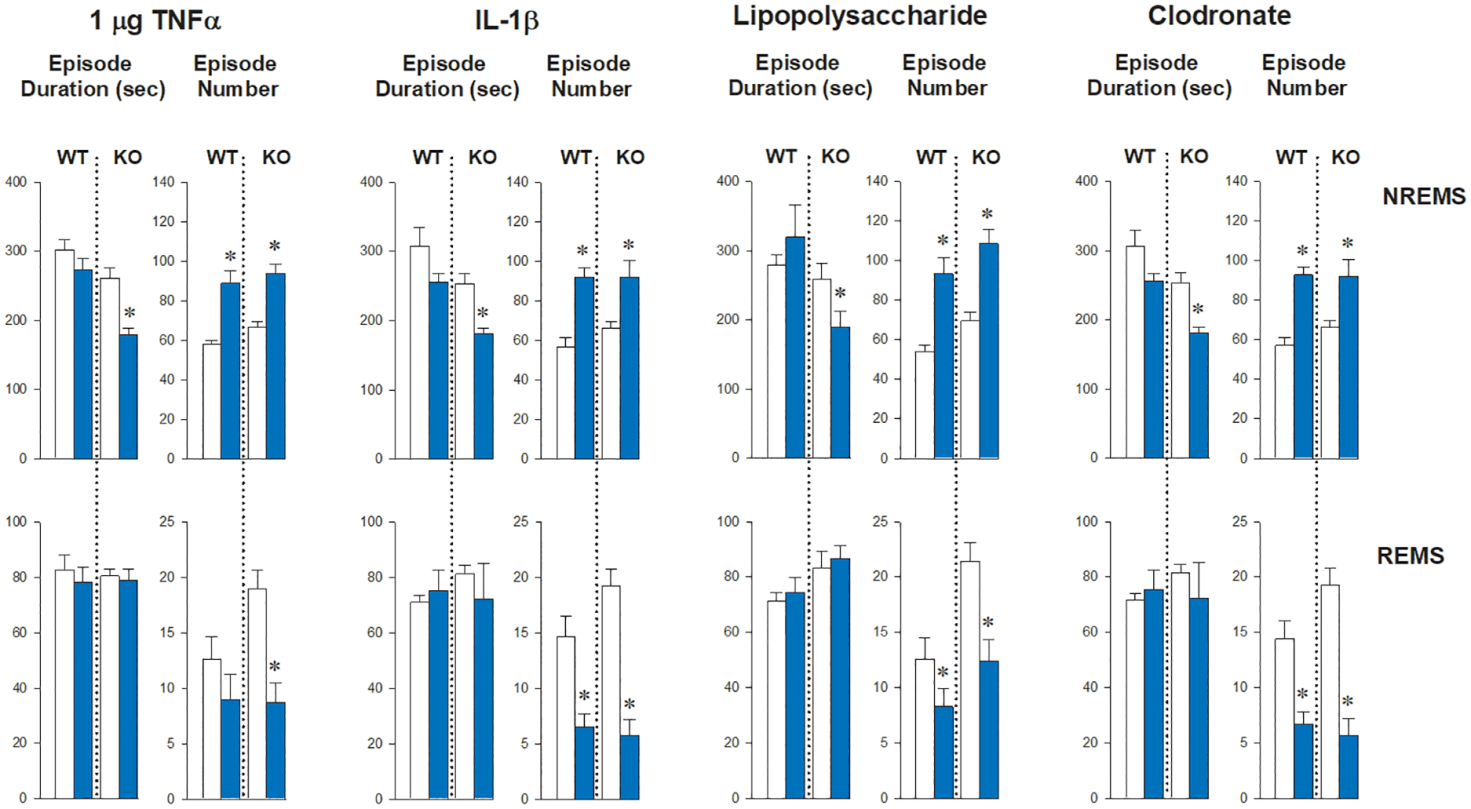
The effects of TNFα, IL-1β, LPS and CCL on the average number and duration of NREMS and REMS episodes in WT and UCP-1 KO mice during the first 12 h after the treatments. White bars: baseline day; blue bars: test day; * significant difference from baseline (paired t-test, p < 0.05); error bar: SE.

Systemic injection of TNFα, IL-1β, LPS and CCL all greatly increased time spent in NREMS in WT mice (Fig 1, S1–S5 Figs, Tables 1–6). The effects were confined to the first 12 h after treatments, i.e., the dark (active) phase of the day. The low dose of TNFα led to an increase of ~1 h while LPS increased NREMS for ~3.5 h over the 12-h dark period. NREMS increases were due to the increased number of NREMS episodes after each treatment (Fig 2). The average duration of the individual NREMS epochs was not affected by any of the inflammatory stimuli.

**Table 1:** Non-rapid-eye movement sleep (NREMS), rapid-eye movement sleep (REMS), body temperature, motor activity and electroencephalographic slow-wave activity (SWA) after 0.3 μg TNFα administration: Statistical results.

0.3 μg TNFα															
	NREMS			REMS			Temperature			Activity			SWA		
	df	F	p	df	F	p	df	F	p	df	F	p	df	F	p
Genotype	1,12	22.9	<0.001	1,12	1.5	n.s.	1,12	26.4	<0.001	1,12	20.3	<0.001	1,12	0.1	n.s.
Treatment	1,12	0.9	n.s.	1,12	4.3	0.058	1,12	0.8	n.s.	1,12	2.9	n.s.	1,12	1.5	n.s.
Treatment x Genotype	1,12	6.7	<0.05	1,12	0.9	n.s.	1,12	0.0	n.s.	1,12	0.9	n.s.	1,12	0.1	n.s.
Treatment x Time	11,132	3.5	<0.001	11,132	1.4	n.s.	11,132	3.5	<0.001	11,132	6.2	<0.001	11,132	2.5	<0.01
Treatment x Time x Genotype	11,132	3.5	<0.001	11,132	0.8	n.s.	11,132	1.8	n.s.	11,132	1.3	n.s.	11,132	0.9	n.s.

**Table 2:** Non-rapid-eye movement sleep (NREMS), rapid-eye movement sleep (REMS), body temperature, motor activity and electroencephalographic slow-wave activity (SWA) after 1 μg TNFα administration: Statistical results.

1 μg TNFα															
	NREMS			REMS			Temperature			Activity			SWA		
	df	F	p	df	F	p	df	F	p	df	F	p	df	F	p
Genotype	1,12	25.3	<0.001	1,12	1.2	n.s.	1,12	7.7	<0.05	1,12	16.3	<0.01	1,12	0.7	n.s.
Treatment	1,12	1.7	n.s.	1,12	0.1	n.s.	1,12	4.5	0.055	1,12	24.5	<0.001	1,12	8.1	<0.05
Treatment x Genotype	1,12	12.8	<0.01	1,12	2.0	n.s.	1,12	7.9	<0.05	1,12	4.0	n.s.	1,12	0.7	n.s.
Treatment x Time	11,132	5.0	<0.001	11,132	7.7	<0.001	11,132	13.4	<0.001	11,132	12.6	<0.001	11,132	9.7	<0.001
Treatment x Time x Genotype	11,132	4.9	<0.001	11,132	1.6	n.s.	11,132	1.8	n.s.	11,132	3.0	<0.01	11,132	0.9	n.s.

**Table 3:** Non-rapid-eye movement sleep (NREMS), rapid-eye movement sleep (REMS), body temperature, motor activity and electroencephalographic slow-wave activity (SWA) after CCL administration: Statistical results.

Clodronate Containing Liposomes															
	NREMS			REMS			Temperature			Activity			SWA		
	df	F	p	df	F	p	df	F	p	df	F	p	df	F	p
Genotype	1,12	32.2	<0.001	1,12	3.1	n.s.	1,12	5.5	<0.05	1,12	4.7	0.051	1,12	0.6	n.s.
Treatment	1,12	1.1	n.s.	1,12	3.8	n.s.	1,12	27.0	<0.001	1,12	72.6	<0.001	1,12	0.9	n.s.
Treatment x Genotype	1,12	5.0	<0.05	1,12	0.5	n.s.	1,12	0.4	n.s.	1,12	3.2	n.s.	1,12	0.6	n.s.
Treatment x Time	11,132	2.8	<0.01	11,132	4.0	<0.001	11,132	4.9	<0.001	11,132	9.0	<0.001	11,132	5.1	<0.001
Treatment x Time x Genotype	11,132	3.3	<0.001	11,132	1.3	n.s.	11,132	1.0	n.s.	11,132	1.5	n.s.	11,132	1.2	n.s.

**Table 4:** Non-rapid-eye movement sleep (NREMS), rapid-eye movement sleep (REMS), body temperature, motor activity and electroencephalographic slow-wave activity (SWA) after LPS administration: Statistical results.

LPS															
	NREMS			REMS			Temperature			Activity			SWA		
	df	F	p	df	F	p	df	F	p	df	F	p	df	F	p
Genotype	1,12	22.9	<0.001	1,12	3.6	n.s.	1,12	0.8	n.s.	1,12	21.3	<0.001	1,12	1.3	n.s.
Treatment	1,12	25.6	<0.001	1,12	6.8	<0.05	1,12	2.7	n.s.	1,12	97.9	<0.001	1,12	10.3	<0.01
Treatment x Genotype	1,12	12.3	<0.001	1,12	1.6	n.s.	1,12	0.5	n.s.	1,12	8.2	<0.05	1,12	1.3	n.s.
Treatment x Time	11,132	9.7	<0.001	11,132	3.6	<0.001	11,132	6.0	<0.001	11,132	17.0	<0.001	11,132	4.3	<0.001
Treatment x Time x Genotype	11,132	6.0	<0.001	11,132	1.0	n.s.	11,132	2.6	<0.01	11,132	2.7	<0.01	11,132	3.4	n.s.

**Table 5:** Non-rapid-eye movement sleep (NREMS), rapid-eye movement sleep (REMS), body temperature, motor activity and electroencephalographic slow-wave activity (SWA) after IL-1β administration: Statistical results.

IL-1β															
	NREMS			REMS			Temperature			Activity			SWA		
	df	F	p	df	F	p	df	F	p	df	F	p	df	F	p
Genotype	1,11	52.1	<0.001	1,11	0.7	n.s.	1,11	3.9	n.s.	1,11	1.0	n.s.	1,11	4.4	n.s.
Treatment	1,11	0.8	n.s.	1,11	4.8	0.051	1,11	7.6	<0.05	1,11	227	<0.001	1,11	44.2	<0.001
Treatment x Genotype	1,11	7.0	<0.05	1,11	2.3	n.s.	1,11	2.3	n.s.	1,11	1.8	n.s.	1,11	4.4	0.059
Treatment x Time	11,121	10.2	<0.001	11,121	4.4	<0.001	11,121	8.9	<0.001	11,121	21.5	<0.001	11,121	32.8	<0.001
Treatment x Time x Genotype	11,121	1.6	n.s.	11,121	0.8	n.s.	11,121	1.4	n.s.	11,121	1.5	n.s.	11,121	3.5	<0.001

n.s.: not significant

**Table 6:** Feeding, oxygen uptake (VO_2_) and respiratory exchange ratio (RER) in response to 1 μg/mouse TNFα: Statistical results.

	Feeding			VO_2_			RER		
	df	F	p	df	F	p	df	F	p
Genotype	1,11	2.2	n.s.	1,12	0.0	n.s.	1,12	3.3	n.s.
Treatment	1,11	254.7	<0.001	1,12	103.8	<0.001	1,12	65.9	<0.001
Treatment x Genotype	1,11	11.2	<0.01	1,12	1.8	n.s.	1,12	1.0	n.s.
Treatment x Time	11,11	62.6	<0.001	1,12	96.1	<0.001	1,12	48.6	<0.001
Treatment x Time x Genotype	11,11	3.4	n.s.	1,12	0.1	n.s.	1,12	1.0	n.s.

The robust NREMS-promoting effects of systemic inflammation observed in WT animals were completely abolished in UCP-1 KO mice (Fig 1, S1–S5 Figs, Tables 1–5). In response to the treatments, UCP-1 KO mice generated increased number of sleep episodes similarly to WTs, but the average duration of the individual NREMS epochs was significantly shorter (Fig 2). As the net outcome of the two opposing factors, total time spent in NREMS remained unchanged.

EEG SWA was suppressed by LPS, IL-1β, and the higher dose of TNFα for 2–10 h after the treatments (S2–S5 Figs). These effects were significantly enhanced in UCP-1 KO mice compared to controls (Tables 1–5).

### The effects of systemic inflammation on REMS

Time spent in REMS was slightly, but significantly, reduced after IL-1β, LPS and CCL injections in WT mice (Fig 1, S1–S5 Figs, Tables 1–5). Similarly to NREMS, decreases in REMS were due to the significantly fewer number of REMS episodes; the average length of REMS epochs was not affected (Fig 2). The effects of IL-1β, LPS and CCL on REMS were not abolished in UCP-1 KO animals. In fact, UCP-1 KO mice showed a significantly enhanced REMS suppression after IL-1β and LPS treatments, as compared to controls.

### The effects of systemic inflammation on body temperature

**Fig 3:**
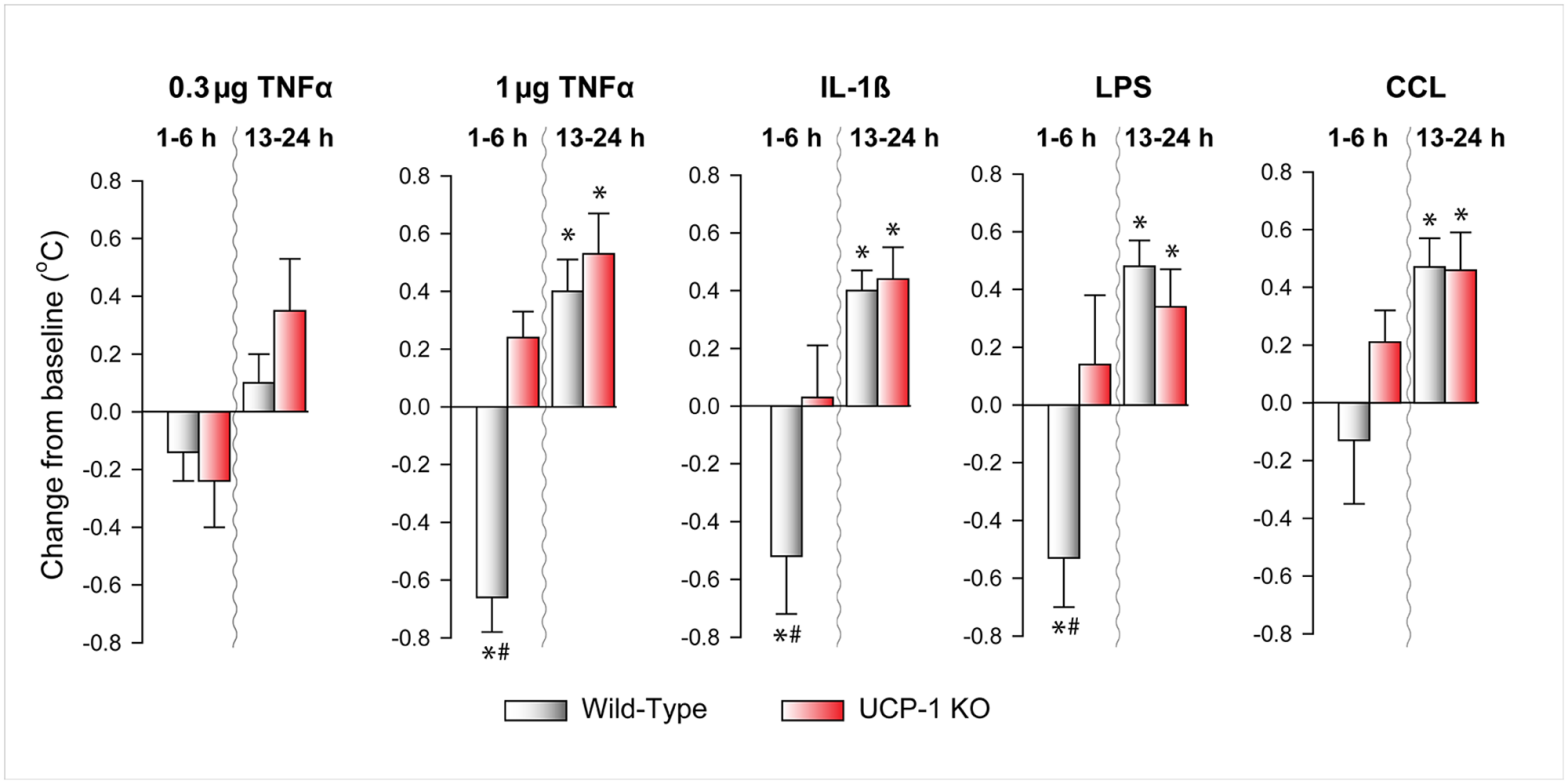
The effects of TNFα, IL-1β, LPS and CCL on body temperature in WT and UCP-1 KO mice. Data are expressed as change of average body temperature during the first 6 h and during hours 13–24 after treatments. See legends to Fig 1 for details.

TNFα, IL-1β and LPS induced biphasic changes in the body temperature of WT animals (Fig 3, S1–S5 Figs, Tables 1–5). An initial hypothermic phase that lasted for 2–10 h was followed by hyperthermia which remained throughout most of the second 12-h period after the treatment (i.e., during the light phase). In response to CCL, there was a clear tendency towards the hypothermic phase, but the change was not significant. The subsequent febrile response was similar to those observed following the other treatments.

Body temperature responses of the UCP-1 KO animals were strikingly different from those of the WTs. The initial hypothermia after TNFα, IL-1β and LPS treatments was completely abolished, while the following hyperthermic phase was not affected.

### The effects of systemic inflammation on motor activity

TNFα, IL-1β, LPS and CCL suppressed spontaneous activity mice (S1–S5 Figs, Tables 1–5). There were no significant differences in the responses of WT and KO animals.

### The effects of TNFα on energy expenditure, respiratory exchange ratio (RER) and feeding

**Fig 4:**
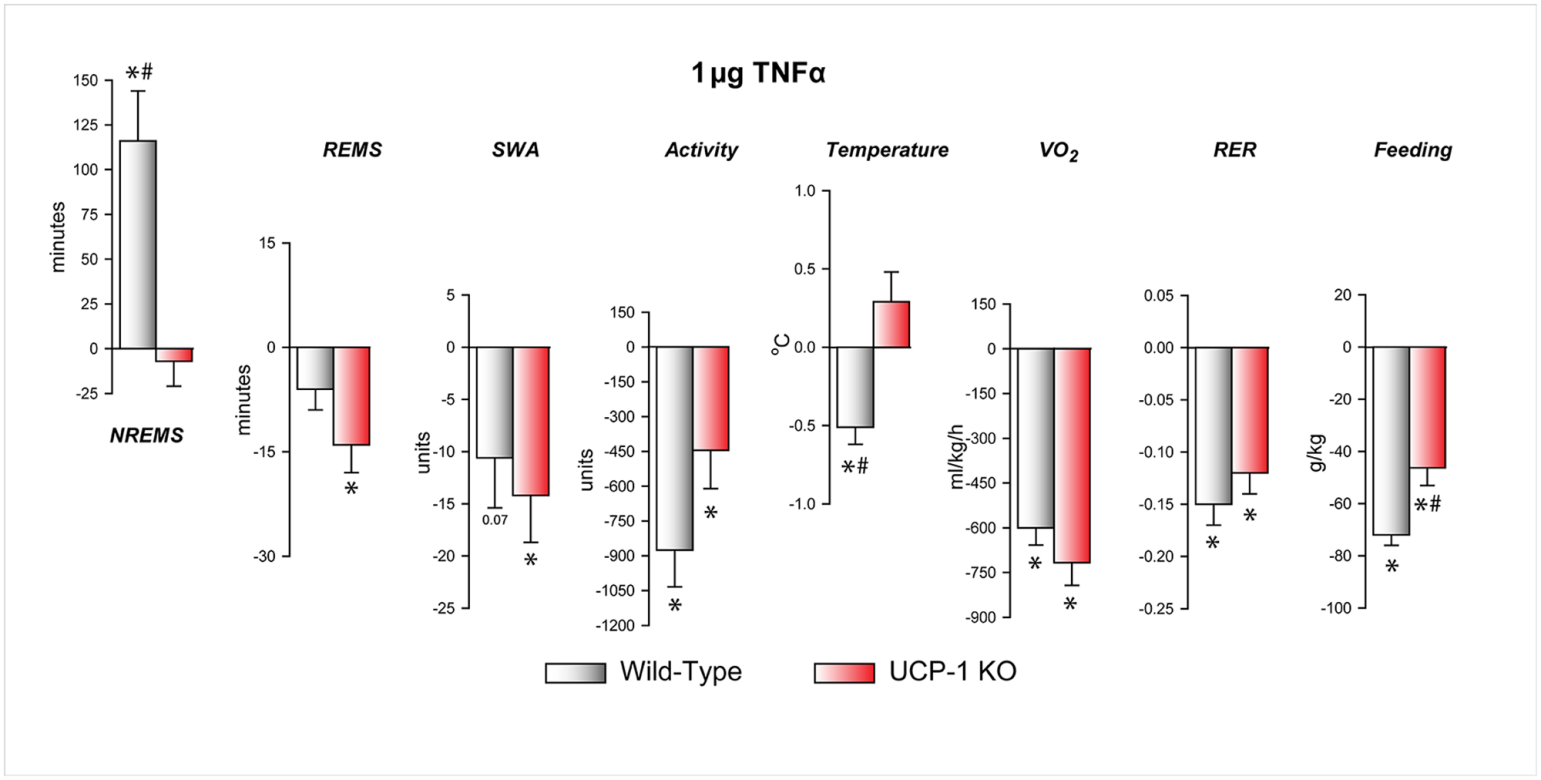
The effects of 1 μg TNFα on NREMS, REMS, slow-wave activity of the electroencephalogram (SWA) body temperature, oxygen uptake (VO_2_), respiratory exchange ratio (RER) and food intake in WT and UCP-1 KO mice. Data are expressed as change from baseline in the first 12 h after treatments. See legends to Fig 1 for details.

Systemic injection of 1 μg TNFα suppressed VO_2_, RER and feeding in both WT and UCP-1 KO mice (Fig 4, S6 Fig, Table 6). Feeding suppression was slightly, but significantly, attenuated in the UCP-1 KO animals.

## Discussion

The major finding of the present study is that systemic inflammation-associated NREMS increases, but not fever, are abolished in UCP-1 KO mice. In WT mice, TNFα, IL-1β, LPS and CCL elicited robust and broadly similar effects on sleep and body temperature. In all four models, NREMS increased in WT mice as reported before [19,27–29]. These responses were completely abolished in UCP-1 KO animals. Since the only recognized function of UCP-1 is to promote thermogenesis by uncoupling ATP formation from mitochondrial respiration in brown adipocytes, the findings suggest that intact BAT thermogenic mechanisms are indispensable for the somnogenic responses to systemic inflammatory stimuli.

The most pronounced phenotypic feature of UCP-1 KO mice is the decreased tolerance to acute cold exposure due to impaired non-shivering thermogenesis [30,31]. UCP-1 KO mice are not obese, feeding is not increased at conventional laboratory temperatures (22–24°C) [30–33], but they become obese when kept at thermoneutral ambient temperature [34]. UCP-1 KO mice have normal VO2 [15,30,32], but the VO2-stimulating effects of β3-AR activation is decreased [15,30,35,36]. BAT mass is increased due to increased deposition of triglycerides [30,35] and mitochondria from UCP-1-ablated mice exhibit a low respiratory rate, *i*.*e*., decreased thermogenesis [33]. Baseline plasma glucose, free fatty acid and insulin levels are normal in UCP-1 KO mice, and they show similar responses to norepinephrine and insulin challenge as the WT controls [35]. Leptin expression in UCP-1 KO mice is not different of the WT animals’ [30], but the lipolytic, feeding-suppressive and energy expenditure increasing activities of exogenously administered leptin are impaired [37–39].

In all four inflammation models, NREMS increases were accompanied by suppressed REMS and biphasic temperature responses as reported previously [22,28,29,40]. Neither REMS decrease nor the hyperthermic phase of the body temperature response was attenuated in UCP-1 KO mice. We conclude that NREMS increase during inflammation is not a consequence of febrile responses and that NREMS and REMS responses are generated by mechanisms that are at least partially independent. Further, our data indicate that the effects of inflammation on sleep and motor activity can be dissociated. Motor activity was suppressed in both genotypes, whereas NREMS was increased only in the WT animals. This suggests that UCP-1 KO mice spent significant time in quiet (immobile) wakefulness after the inflammatory challenges. This also underlies the notion that motor activity is not a reliable measure of sleep-wake activity in mice and possibly in other species.

The amount of sleep in mice is the function of the number of sleep episodes and their average duration. Episode number reflects the ability of the organism to initiate sleep, while the duration of the episodes is considered as a measure of the ability to maintain sleep once it is triggered. Inflammatory stimuli in WT mice affected the incidence of sleep episodes, i.e., the number of occurrences of NREMS and REMS epochs. Time spent in NREMS was increased due to the increased number of NREMS epochs, while REMS was suppressed due to a decrease in the epoch number. The episode durations for the two sleep stages were unaltered. This is in line with prior findings on the sleep architecture after systemic LPS and TNFα treatments in mice [41]. In UCP-1 KO mice, NREMS episode numbers were still elevated in response to inflammatory stimuli, but the animals were unable to maintain sleep to the same extent as the WTs. This suggests that signals arising from BAT are needed for the maintenance and not for initiation of NREMS during inflammation. Inflammation suppressed the incidence, but not the average duration, of REMS episodes. This effect was still present in UCP-1 KO mice indicating that the maintenance of REMS is efficient, even during the course of systemic inflammation.

EEG SWA under normal conditions is regarded as a measure of sleep intensity. It can change because sleep need changes, e.g., after sleep deprivation, but it can also change independently of sleep pressure (reviewed in [42]). Our results are consistent with previous observations that in mice TNFα, LPS and IL-1β suppress EEG SWA [28,41]. In other species, e.g., rabbits and rats, EEG SWA is increased in response to proinflammatory cytokines [43,44]. This demonstrates that changes in SWA are species-specific and EEG SWA responses are not generalizable attributes of systemic inflammation. EEG SWA responses in UCP-1 KO animals were accentuated suggesting that UCP-1-dependent thermogenesis is important not only for increased NREMS during systemic inflammation but also modulates the EEG manifestation that accompanies increased sleep.

Systemic administration of TNFα, IL-1β and LPS activates various markers of BAT thermogenic activity. For example, systemic injection of TNFα stimulates sympathetic outflow to BAT and enhances thermogenic activity as assessed by mitochondrial cGMP-binding [10], stimulates fatty acid synthesis in brown adipocytes [45] and stimulates UCP-1 mRNA expression in BAT [13]. Intracerebroventricular injection of TNFα also stimulates mitochondrial GDP-binding [46,47] and UCP-1 mRNA expression [48] in brown fat. Similarly, systemic or icv administration of IL-1β stimulates GDP-binding in brown adipocytes [11,47], enhances BAT blood flow [11] and enhances cold-induced sympathetic outflow to BAT [49]. Systemic administration of LPS elicits increases in BAT temperature [50–52], stimulates GDP-binding [12] and increases UCP-1 mRNA expression in BAT [53].

Proinflammatory stimuli may act on peripheral and/or central target sites to stimulate BAT. One possibility is that proinflammatory cytokines act directly on brown adipocytes to stimulate their thermogenic activity. The findings that cytokines have a general suppressive effect on cultured brown adipocytes [54–56] seem to refute this possibility, although only limited conclusions can be drawn regarding the direct *in vivo* effects of bolus injections of proinflammatory agents from studies when brown adipocytes were incubated with high concentrations of cytokines for an extended period of time *in vitro*. Another possibility is that proinflammatory stimuli activate vagus afferents to trigger a reflex that leads to BAT activation. Prior findings that subdiaphragmal vagotomy also attenuates sleep responses induced by systemically administered IL-1β, TNFα and LPS are in line with this possibility [18,21,43,57]. This mechanism assumes that activation of sensory vagus neurons in fact leads to BAT thermogenesis. Several [58–61], but not all [62], studies support the role of vagus afferents in stimulating BAT activity. A third possibility is that proinflammatory signals activate BAT via indirect non-neuronal peripheral mechanisms. TNFα, IL-1β and LPS stimulate lipolysis resulting in high levels of circulating free fatty acids (reviewed in [63]). Fatty acids are potent activators of UCP-1 and BAT thermogenesis [9]. Finally, in addition to acting on peripheral targets, circulating cytokines may gain direct access to central circuits that are involved in the regulation of BAT activity. Centrally acting IL-1β or TNFα stimulates BAT activity in rats [11,46,48].

Activation of UCP-1-dependent processes in BAT is not necessarily manifested in significant increases in body temperature. Our findings that TNFα, IL-1β, LPS and CCL administration elicited a prompt hypothermic response simultaneously with the UCP-1-dependent NREMS increases support this notion. Previously we described that sensory innervation of the BAT is responsible, at least in part, for the UCP-1-dependent sleep responses [14]. It is possible that proinflammatory stimuli are sufficient to stimulate BAT thermogenesis to the extent that thermosensitive afferents become activated, but it is insufficient to elicit robust BAT thermogenesis that may lead to an overall increase in core body temperature.

The phenomenon that proinflammatory stimuli produce biphasic temperature responses in mice, an initial drop in body temperature followed by hyperthermia, is well documented [26,40,64]. Our observation that it is the initial hypothermic phase of the effects of proinflammatory stimuli that are abolished in UCP-1 KO animals is intriguing. There are several possible mechanisms that could explain this finding. One possibility is that UCP-1 KO animals develop a compensatory mechanism for non-shivering thermogenesis through other types of uncoupling proteins. For example, UCP-3 is expressed predominantly in skeletal muscle [65], and it appears to be sufficiently thermogenic under certain conditions [66,67]. Possibly, UCP-1-independent activation of this, or a similar, mechanism could mask the initial hypothermic phase.

BAT heat production is a graded response, and if the thermogenic activity of BAT is robust enough, it will be manifested as increased core body temperature. It is also possible that various stimuli, including inflammatory signals, activate BAT thermogenesis sufficiently to increase local BAT temperature, but it is not sufficient to affect core body temperature. A subtle increase in BAT temperature may activate local thermoreceptors, which, in turn, may trigger a negative thermoregulatory feedback leading to an initial drop in body temperature. Several lines of evidence support this possibility. One, there is evidence that various processes linked to inflammation are under the control of inhibitory neural reflex mechanisms [68]. In fact, it has been demonstrated that the hypothermic phase after pro-inflammatory challenges is a regulated decrease in body temperature [69–71]. Two, temperature-sensing transient receptor potential vanilloid 4 (TRPV4) channels are expressed in BAT [72,73]. TRPV4 channel activation occurs in a physiological temperature range, thus could potentially detect subtle changes in BAT temperature [74]. In line with the presence of thermoreceptors in BAT, it has been shown that a population of BAT afferents is temperature sensitive [75]. Three, data suggest that sensory nerve fibers in interscapular BAT participate in a feedback loop to prevent overheating of the tissue caused by local thermogenesis [14,75], and sensory feedback from BAT plays a role in thermoregulation [76]. It is possible that proinflammatory signals stimulate UCP-1-dependent thermogenesis in BAT, which, in turn, activates thermo-sensitive afferents. The activation of the reflex may result in the recruitment of thermolytic mechanisms and the suppression of thermogenesis. This process could potentially lead to hypothermia in WT mice. In the absence of UCP-1, the reflex is not activated and thus the initial hypothermic response is abolished.

BAT afferents not only play a potential role in thermoregulation, but they are also key players in BAT activation-induced sleep responses. In previous studies we found that sleep loss induced increased expression of UCP-1 mRNA in BAT, and recovery sleep responses to sleep loss were significantly attenuated in UCP-1 KO mice. The same phenotype was reproduced in WT mice with the sensory denervation of BAT [14]. This indicates that both UCP-1 and the intact afferentation arising from BAT are necessary for recovery responses after sleep loss. We speculate that local, UCP-1-dependent production of metabolites or thermic signals activate BAT afferents, and the somnogenic signals are carried by ascending spinal pathways to sleep-regulatory centers in the brain.

The mechanism of fever that accompanies inflammation is subject of debate. Early studies showed increased BAT temperature in response to endotoxin administration and the general conclusion was that BAT plays a role in the development of fever [50,51,77]. Subsequent studies confirmed that proinflammatory stimuli may activate BAT as measured by GDP-binding and UCP-1 expression [10–13], but the extent to which this activation is in fact a significant factor in the development of fever has not been clearly established. In our study, the delayed hyperthermic responses to IL-1β, TNFα, LPS and CCL, which develop ~12 h after the treatments, remained unchanged in UCP-1 KO mice. This indicates that the response, which is generally considered fever, is independent of BAT thermogenesis. This is consistent with prior observations that UCP-1-dependent thermogenesis is dispensable for IL-1β- and LPS-induced fever [67,78]. The finding that VO_2_ was not elevated during the second 12 h after TNFα administration suggests that the increased body temperature during this period is not due to increased heat production, but to the suppression of heat-loss mechanisms and elevated heat conservation.

Metabolism, inflammation and sleep are intricately connected. Metabolic and proinflammatory signals affect the amount and quality of sleep; sleep or the lack thereof has profound effects on whole body metabolism [79]. With the emergence of BAT as a significant player in the regulation of metabolism in adult humans (reviewed in [80]), the role of BAT at the intersection of sleep and metabolic regulation gained renewed significance [14,15]. In the present study we demonstrated that a broad range of inflammatory stimuli facilitate NREMS and elicit biphasic temperature responses in WT mice. The sleep increase and the hypothermic phase of the thermoregulatory response were abolished in UCP-1 KO animals. The finding that functional UCP-1 is key to sleep responses associated with systemic inflammation, but dispensable for febrile responses may open new perspective in the treatment of inflammation-related pathologies.

## Supporting information

S1 Fig The effects of 0.3 μg/mouse TNFα on the amount of wakefulness, NREMS, REMS, SWA, body temperature and activity in WT and UCP-1 KO mice. Data are averaged in 2-h blocks. Shaded area: dark phase of the day; * significant difference from baseline (SNK test); error bars: SE.
(DOCX)

S2 Fig The effects of 1 μg/mouse TNFα on the amount of wakefulness, NREMS, REMS, SWA, body temperature and activity in WT and UCP-1 KO mice. See legends to S1 Fig for details.
(DOCX)

S3 Fig The effects of 0.4 μg/mouse IL-1β on the amount of wakefulness, NREMS, REMS, SWA, body temperature and activity in WT and UCP-1 KO mice. See legends to S1 Fig for details.
(DOCX)

S4 Fig The effects of LPS on the amount of wakefulness, NREMS, REMS, SWA, body temperature and activity in WT and UCP-1 KO mice. See legends to S1 Fig for details.
(DOCX)

S5 Fig The effects of CCL on the amount of wakefulness, NREMS, REMS, SWA, body temperature and activity in WT and UCP-1 KO mice. See legends to S1 Fig for details.
(DOCX)

S6 Fig The effects of 1 μg/mouse TNFα on VO_2_, RER and food intake. See legends to S1 Fig for details.
(DOCX)

## References

[1] Copinschi G, Leproult R, Spiegel K. The important role of sleep in metabolism. Front Horm Res 2014;42:59–72. doi: 10.1159/000358858 24732925 10.1159/000358858

[2] Krueger JM, Majde JA, Obál F. Sleep in host defense. Brain Behav Immun 2003;17 Suppl 1:S41–S47. 12615185 10.1016/s0889-1591(02)00065-x

[3] Shukla C, Basheer R. Metabolic signals in sleep regulation: recent insights. Nat Sci Sleep 2016;8:9–20. doi: 10.2147/NSS.S62365 26793010 10.2147/NSS.S62365 PMC4708875

[4] Simpson N, Dinges DF. Sleep and inflammation. Nutr Rev 2007;65:S244–S252. 18240557 10.1111/j.1753-4887.2007.tb00371.x

[5] Hotamisligil GS. Inflammation and metabolic disorders. Nature 2006;444:860–7. doi: 10.1038/nature05485 17167474 10.1038/nature05485

[6] Weisberg SP, McCann D, Desai M, Rosenbaum M, Leibel RL, Ferrante AW Jr. Obesity is associated with macrophage accumulation in adipose tissue. J Clin Invest 2003;112:1796–808. doi: 10.1172/JCI19246 14679176 10.1172/JCI19246 PMC296995

[7] Wisse BE. The inflammatory syndrome: the role of adipose tissue cytokines in metabolic disorders linked to obesity. J Am Soc Nephrol 2004;15:2792–800. doi: 10.1097/01.ASN.0000141966.69934.21 15504932 10.1097/01.ASN.0000141966.69934.21

[8] Cannon B, Nedergaard J. Brown adipose tissue: function and physiological significance. Physiol Rev 2004;84:277–359. doi: 10.1152/physrev.00015.2003 14715917 10.1152/physrev.00015.2003

[9] Crichton PG, Lee Y, Kunji ER. The molecular features of uncoupling protein 1 support a conventional mitochondrial carrier-like mechanism. Biochimie 2017;134:35–50. doi: 10.1016/j.biochi.2016.12.016 28057583 10.1016/j.biochi.2016.12.016 PMC5395090

[10] Coombes RC, Rothwell NJ, Shah P, Stock MJ. Changes in thermogenesis and brown fat activity in response to tumour necrosis factor in the rat. Biosci Rep 1987;7:791–9. 3447641 10.1007/BF01116752

[11] Dascombe MJ, Rothwell NJ, Sagay BO, Stock MJ. Pyrogenic and thermogenic effects of interleukin 1β in the rat. Am J Physiol 1989;256:E7–11. doi: 10.1152/ajpendo.1989.256.1.E7 2783533 10.1152/ajpendo.1989.256.1.E7

[12] Jepson MM, Millward DJ, Rothwell NJ, Stock MJ. Involvement of sympathetic nervous system and brown fat in endotoxin-induced fever in rats. Am J Physiol 1988;255:E617–E620. doi: 10.1152/ajpendo.1988.255.5.E617 3056031 10.1152/ajpendo.1988.255.5.E617

[13] Masaki T, Yoshimatsu H, Chiba S, Hidaka S, Tajima D, Kakuma T, et al Tumor necrosis factor-alpha regulates in vivo expression of the rat UCP family differentially. Biochim Biophys Acta 1999;1436:585–92. 9989288 10.1016/s0005-2760(98)00173-8

[14] Szentirmai É, Kapás L. Intact brown adipose tissue thermogenesis is required for restorative sleep responses after sleep loss. Eur J Neurosci 2014;39:984–98. doi: 10.1111/ejn.12463 24372950 10.1111/ejn.12463

[15] Szentirmai E, Kapás L. The role of the brown adipose tissue in beta3-adrenergic receptor activation-induced sleep, metabolic and feeding responses. Sci Rep 2017;7:958 doi: 10.1038/s41598-017-01047-1 28424466 10.1038/s41598-017-01047-1 PMC5430421

[16] Konsman JP, Parnet P, Dantzer R. Cytokine-induced sickness behaviour: mechanisms and implications. Trends Neurosci 2002;25:154–9. 11852148 10.1016/s0166-2236(00)02088-9

[17] Szelenyi Z, Szekely M. Sickness behavior in fever and hypothermia. Front Biosci 2004;9:2447–56. 15353297 10.2741/1406

[18] Kapás L, Hansen MK, Chang HY, Krueger JM. Vagotomy attenuates but does not prevent the somnogenic and febrile effects of lipopolysaccharide in rats. Am J Physiol 1998;274:R406–R411. 9486298 10.1152/ajpregu.1998.274.2.R406

[19] Morrow JD, Opp MR. Diurnal variation of lipopolysaccharide-induced alterations in sleep and body temperature of interleukin-6-deficient mice. Brain Behav Immun 2005;19:40–51. doi: 10.1016/j.bbi.2004.04.001 15581737 10.1016/j.bbi.2004.04.001

[20] Nadjar A, Blutstein T, Aubert A, Laye S, Haydon PG. Astrocyte-derived adenosine modulates increased sleep pressure during inflammatory response. Glia 2013;61:724–31. doi: 10.1002/glia.22465 23378051 10.1002/glia.22465

[21] Opp MR, Toth LA. Somnogenic and pyrogenic effects of interleukin-1beta and lipopolysaccharide in intact and vagotomized rats. Life Sci 1998;62:923–36. 9496715 10.1016/s0024-3205(98)00010-1

[22] Szentirmai É, Krueger JM. Sickness behaviour after lipopolysaccharide treatment in ghrelin deficient mice. Brain Behav Immun 2014;36:200–6. doi: 10.1016/j.bbi.2013.11.017 24309634 10.1016/j.bbi.2013.11.017 PMC3951816

[23] Kelley KW, Bluthé RM, Dantzer R, Zhou JH, Shen WH, Johnson RW, et al Cytokine-induced sickness behavior. Brain Behav. Immun. 2003;17:S112–S118. 12615196 10.1016/s0889-1591(02)00077-6

[24] Van Rooijen N, Bakker J, Sanders A. Transient suppression of macrophage functions by liposome-encapsulated drugs. Trends Biotechnol 1997;15:178–85. 9161052 10.1016/s0167-7799(97)01019-6

[25] Van Rooijen N, Sanders A. Liposome mediated depletion of macrophages: mechanism of action, preparation of liposomes and applications. J Immunol Methods 1994;174:83–93. 8083541 10.1016/0022-1759(94)90012-4

[26] Ames C, Boland E, Szentirmai É. Effects of macrophage depletion on sleep in mice. PLoS One 2016;11:e0159812 doi: 10.1371/journal.pone.0159812 27442442 10.1371/journal.pone.0159812 PMC4956207

[27] Fang J, Wang Y, Krueger JM. Mice lacking the TNF 55 kDa receptor fail to sleep more after TNFα treatment. J Neurosci 1997;17:5949–55. 9221791 10.1523/JNEUROSCI.17-15-05949.1997 PMC6573218

[28] Fang J, Wang Y, Krueger JM. Effects of interleukin-1β on sleep are mediated by the type I receptor. Am J Physiol 1998;274:R655–R660. 9530230 10.1152/ajpregu.1998.274.3.R655

[29] Toth LA, Opp MR. Cytokine- and microbially induced sleep responses of interleukin-10 deficient mice. Am J Physiol Regul Integr Comp Physiol 2001;280:R1806–R1814. doi: 10.1152/ajpregu.2001.280.6.R1806 11353686 10.1152/ajpregu.2001.280.6.R1806

[30] Enerbäck S, Jacobsson A, Simpson EM, Guerra C, Yamashita H, Harper M, et al Mice lacking mitochondrial uncoupling protein are cold-sensitive but not obese. Nature 1997;387:90–94. 10.1038/387090a0 9139827

[31] Ukropec J, Anunciado RP, Ravussin Y, Hulver MW, Kozak LP. UCP1-independent thermogenesis in white adipose tissue of cold-acclimated Ucp1-/- mice. J Biol Chem 2006;281:31894–908. doi: 10.1074/jbc.M606114200 16914547 10.1074/jbc.M606114200

[32] Liu X, Rossmeisl M, McClaine J, Kozak LP. Paradoxical resistance to diet-induced obesity in UCP1-deficient mice. J Clin Invest 2003;111:399–407. doi: 10.1172/JCI15737 12569166 10.1172/JCI15737 PMC151850

[33] Matthias A, Jacobsson A, Cannon B, Nedergaard J. The bioenergetics of brown fat mitochondria from UCP1-ablated mice. J Biol Chem 1999;274:28150–60. 10497167 10.1074/jbc.274.40.28150

[34] Feldmann HM, Golozoubova V, Cannon B, Nedergaard J. UCP1 ablation induces obesity and abolishes diet-induced thermogenesis in mice exempt from thermal stress by living at thermoneutrality. Cell Metab 2009;9:203–209. doi: 10.1016/j.cmet.2008.12.014 19187776 10.1016/j.cmet.2008.12.014

[35] Inokuma K, Ogura-Okamatsu Y, Toda C, Kimura K, Yamashita H, Saito M. Uncoupling protein 1 is necessary for norepinephrine-induced glucose utilization in brown adipose tissue. Diabetes 2005;54:1385–91. 15855324 10.2337/diabetes.54.5.1385

[36] Okamatsu-Ogura Y, Kitao N, Kimura K, Saito M. Brown fat UCP1 is not involved in the febrile and thermogenic responses to IL-1β in mice. Am J Physiol Endocrinol Metab 2007;292:E1135–39. doi: 10.1152/ajpendo.00425.2006 17164436 10.1152/ajpendo.00425.2006

[37] Commins SP, Watson PM, Frampton IC, Gettys TW. Leptin selectively reduces white adipose tissue in mice via a UCP1-dependent mechanism in brown adipose tissue. Am J Physiol Endocrinol Metab 2001;280:E373–7. 10.1152/ajpendo.2001.280.2.E372 11158943

[38] Okamatsu-Ogura Y, Uozumi A, Toda C, Kimura K, Yamashita H, Saito M. Uncoupling protein 1 contributes to fat-reducing effect of leptin. Obes Res Clin Pract 2007;1:233–41. 10.1016/j.orcp.2007.08.001 24351582

[39] Okamatsu-Ogura Y, Nio-Kobayashi J, Iwanaga T, Terao A, Kimura K, Saito M. Possible involvement of uncoupling protein 1 in appetite control by leptin. Exp Biol Med 2011;236:1274–81. 10.1258/ebm.2011.011143 21987829

[40] Wang J, Ando T, Dunn AJ. Effect of homologous interleukin-1, interleukin-6 and tumor necrosis factor-alpha on the core body temperature of mice. Neuroimmunomodulation 1997;4:230–6. doi: 10.1159/000097341 9650815 10.1159/000097341

[41] Zielinski MR, Dunbrasky DL, Taishi P, Souza G, Krueger JM. Vagotomy attenuates brain cytokines and sleep induced by peripherally administered tumor necrosis factor-alpha and lipopolysaccharide in mice. Sleep 2013;36:1227–38. doi: 10.5665/sleep.2892 23904683 10.5665/sleep.2892 PMC3700720

[42] Davis CJ, Clinton JM, Jewett KA, Zielinski MR, Krueger JM. Delta wave power: an independent sleep phenotype or epiphenomenon? J Clin Sleep Med 2011;7(5 Suppl):S16–8 doi: 10.5664/JCSM.1346 22003323 10.5664/JCSM.1346 PMC3190419

[43] Kubota T, Fang J, Guan Z, Brown RA, Krueger JM. Vagotomy attenuates tumor necrosis factor-alpha-induced sleep and EEG delta-activity in rats. Am J Physiol Regul Integr Comp Physiol 2001;280:R1213–20. doi: 10.1152/ajpregu.2001.280.4.R1213 11247847 10.1152/ajpregu.2001.280.4.R1213

[44] Shoham S, Davenne D, Cady AB, Dinarello CA, Krueger JM. Recombinant tumor necrosis factor and interleukin 1 enhance slow-wave sleep. Am J Physiol 1987;253:R142–9. doi: 10.1152/ajpregu.1987.253.1.R142 3496800 10.1152/ajpregu.1987.253.1.R142

[45] Lopez-Soriano J, Argiles JM, Lopez-Soriano FJ. Metabolic effects of tumour necrosis factor-α on rat brown adipose tissue. Mol Cell Biochem 1995;143:113–8. 7596346 10.1007/BF01816944

[46] Rothwell NJ. Central effects of TNFα on thermogenesis and fever in the rat. Biosci Rep 1988;8:345–52. 3263887 10.1007/BF01115225

[47] Rothwell NJ. CRF is involved in the pyrogenic and thermogenic effects of interleukin 1β in the rat. Am J Physiol Endocrinol Metab 1989;256:E111–5. 10.1152/ajpendo.1989.256.1.E111 2783532

[48] Arruda AP, Milanski M, Romanatto T, Solon C, Coope A, Alberici LC, et al Hypothalamic actions of tumor necrosis factor alpha provide the thermogenic core for the wastage syndrome in cachexia. Endocrinology 2010;151:683–94. doi: 10.1210/en.2009-0865 19996183 10.1210/en.2009-0865

[49] Kenney MJ, Blecha F, Morgan DA, Fels RJ. Interleukin-1β alters brown adipose tissue but not renal sympathetic nerve responses to hypothermia. Am J Physiol Heart Circ Physiol 2001;281:H2441–5. doi: 10.1152/ajpheart.2001.281.6.H2441 11709410 10.1152/ajpheart.2001.281.6.H2441

[50] Székely M, Szelényi Z, Sümegi I. Brown adipose tissue as a source of heat during pyrogen-induced fever. Acta Physiol Acad Sci Hung 1973;43:85–8. 4589011

[51] Blatteis CM. Effect of propranolol on endotoxin-induced pyrogenesis in newborn and adult guinea pigs. J Appl Physiol 1976;40:35–9. doi: 10.1152/jappl.1976.40.1.35 765316 10.1152/jappl.1976.40.1.35

[52] Székely M, Szelényi Z. Endotoxin fever in the rat. Acta Physiol Acad Sci Hung 1979;53:265–77. 396758

[53] Borges BC, Rorato R, Uchoa ET, Marangon P, da Silva GS, de Paula FJ, et al High-fat diet induces site-specific unresponsiveness to LPS-stimulated STAT3 activation in the hypothalamus. Am J Physiol Regul Integr Comp Physiol 2014;306:R34–R44. doi: 10.1152/ajpregu.00147.2013 24226027 10.1152/ajpregu.00147.2013

[54] Mracek T, Cannon B, Houstek J. IL-1 and LPS but not IL-6 inhibit differentiation and downregulate PPAR gamma in brown adipocytes. Cytokine 2004;26:9–15. doi: 10.1016/j.cyto.2003.12.001 15016406 10.1016/j.cyto.2003.12.001

[55] Nisoli E, Briscini L, Tonello C, De Giuli-Morghen C, Carruba MO. Tumor necrosis factor-alpha induces apoptosis in rat brown adipocytes. Cell Death Differ 1997;4:771–8. doi: 10.1038/sj.cdd.4400292 16465289 10.1038/sj.cdd.4400292

[56] Porras A, Alvarez AM, Valladares A, Benito M. TNF-α induces apoptosis in rat fetal brown adipocytes in primary culture. FEBS Lett 1997;416:324–8. 9373178 10.1016/s0014-5793(97)01204-0

[57] Hansen MK, Krueger JM. Subdiaphragmatic vagotomy blocks the sleep- and fever-promoting effects of interleukin-1β. Am J Physiol 1997;273:R1246–R1253. 9362287 10.1152/ajpregu.1997.273.4.R1246

[58] Blouet C, Schwartz GJ. Duodenal lipid sensing activates vagal afferents to regulate non-shivering brown fat thermogenesis in rats. PLoS One 2012;7:e51898 doi: 10.1371/journal.pone.0051898 23251649 10.1371/journal.pone.0051898 PMC3522613

[59] Ono K, Tsukamoto-Yasui M, Hara-Kimura Y, Inoue N, Nogusa Y, Okabe Y, et al Intragastric administration of capsiate, a transient receptor potential channel agonist, triggers thermogenic sympathetic responses. J Appl Physiol 2011;110:789–98. doi: 10.1152/japplphysiol.00128.2010 21071592 10.1152/japplphysiol.00128.2010

[60] Sakaguchi T, Yamazaki M. Hepatic portal injection of glucose elevates efferent sympathetic discharges of interscapular brown adipose tissue. Exp Neurol 1988;101:464–9. 3416988 10.1016/0014-4886(88)90057-x

[61] Vijgen GH, Bouvy ND, Leenen L, Rijkers K, Cornips E, Majoie M, et al Vagus nerve stimulation increases energy expenditure: relation to brown adipose tissue activity. PLoS One 2013;8:e77221 doi: 10.1371/journal.pone.0077221 24194874 10.1371/journal.pone.0077221 PMC3806746

[62] Madden CJ, Santos da Conceicao EP, Morrison SF. Vagal afferent activation decreases brown adipose tissue (BAT) sympathetic nerve activity and BAT thermogenesis. Temperature (Austin) 2017;4:89–96. 28349097 10.1080/23328940.2016.1257407 PMC5356211

[63] Grunfeld C, Feingold KR. Regulation of lipid metabolism by cytokines during host defense. Nutrition 1996;12:S24–S26. 8850215 10.1016/0899-9007(96)90013-1

[64] Kozak W, Conn CA, Kluger MJ. Lipopolysaccharide induces fever and depresses locomotor activity in unrestrained mice. Am J Physiol 1994;266:R125–R135. doi: 10.1152/ajpregu.1994.266.1.R125 8304533 10.1152/ajpregu.1994.266.1.R125

[65] Vidal-Puig A, Solanes G, Grujic D, Flier JS, Lowell BB. UCP3: an uncoupling protein homologue expressed preferentially and abundantly in skeletal muscle and brown adipose tissue. Biochem Biophys Res Commun 1997;235:79–82. doi: 10.1006/bbrc.1997.6740 9196039 10.1006/bbrc.1997.6740

[66] Mills EM, Banks ML, Sprague JE, Finkel T. Pharmacology: uncoupling the agony from ecstasy. Nature 2003;426:403–4. doi: 10.1038/426403a 14647371 10.1038/426403a

[67] Riley CL, Dao C, Kenaston MA, Muto L, Kohno S, Nowinski SM, et al The complementary and divergent roles of uncoupling proteins 1 and 3 in thermoregulation. J Physiol 2016;594:7455–64. doi: 10.1113/JP272971 27647490 10.1113/JP272971 PMC5157057

[68] Tracey KJ. The inflammatory reflex. Nature 2002;420:853–9. doi: 10.1038/nature01321 12490958 10.1038/nature01321

[69] Derijk RH, Van KM, Van RN, Berkenbosch F. Hypothermia to endotoxin involves reduced thermogenesis, macrophage-dependent mechanisms, and prostaglandins. Am J Physiol 1994;266:R1–R8. doi: 10.1152/ajpregu.1994.266.1.R1 8304529 10.1152/ajpregu.1994.266.1.R1

[70] Dogan MD, Ataoglu H, Akarsu ES. Characterization of the hypothermic component of LPS-induced dual thermoregulatory response in rats. Pharmacol Biochem Behav 2002;72:143–50. 11900781 10.1016/s0091-3057(01)00736-5

[71] Romanovsky AA, Shido O, Sakurada S, Sugimoto N, Nagasaka T. Endotoxin shock: thermoregulatory mechanisms. Am J Physiol 1996;270:R693–R703. doi: 10.1152/ajpregu.1996.270.4.R693 8967396 10.1152/ajpregu.1996.270.4.R693

[72] Wang T, Wang Y, Yamashita H. Evodiamine inhibits adipogenesis via the EGFR-PKCα-ERK signaling pathway. FEBS Lett 2009;583:3655–9. doi: 10.1016/j.febslet.2009.10.046 19854188 10.1016/j.febslet.2009.10.046

[73] Ye L, Kleiner S, Wu J, Sah R, Gupta RK, Banks AS, et al TRPV4 is a regulator of adipose oxidative metabolism, inflammation, and energy homeostasis. Cell 2012;151:96–110. doi: 10.1016/j.cell.2012.08.034 23021218 10.1016/j.cell.2012.08.034 PMC3477522

[74] Watanabe H, Vriens J, Suh SH, Benham CD, Droogmans G, Nilius B. Heat-evoked activation of TRPV4 channels in a HEK293 cell expression system and in native mouse aorta endothelial cells. J Biol Chem 2002;277:47044–51. doi: 10.1074/jbc.M208277200 12354759 10.1074/jbc.M208277200

[75] Osaka T, Kobayashi A, Namba Y, Ezaki O, Inoue S, Kimura S, et al Temperature- and capsaicin-sensitive nerve fibers in brown adipose tissue attenuate thermogenesis in the rat. Pflugers Arch 1998;437:36–42. doi: 10.1007/s004240050743 9817783 10.1007/s004240050743

[76] Vaughan CH, Bartness TJ. Anterograde transneuronal viral tract tracing reveals central sensory circuits from brown fat and sensory denervation alters its thermogenic responses. Am J Physiol Regul Integr Comp Physiol 2012;302:R1049–R1058. doi: 10.1152/ajpregu.00640.2011 22378771 10.1152/ajpregu.00640.2011 PMC3362143

[77] Székely M, Szolcsányi J. Endotoxin fever in capsaicin treated rats. Acta Physiol Acad Sci Hung 1979;53:469–77. 397719

[78] Okamatsu-Ogura Y, Kitao N, Kimura K, Saito M. Brown fat UCP1 is not involved in the febrile and thermogenic responses to IL-1beta in mice. Am J Physiol Endocrinol Metab 2007;292:E1135–E1139. doi: 10.1152/ajpendo.00425.2006 17164436 10.1152/ajpendo.00425.2006

[79] Schmid SM, Hallschmid M, Schultes B. The metabolic burden of sleep loss. Lancet Diabetes Endocrinol 2015;3:52–62. doi: 10.1016/S2213-8587(14)70012-9 24731536 10.1016/S2213-8587(14)70012-9

[80] Betz MJ, Enerbäck S. Human brown adipose tissue: what we have learned so far. Diabetes 2015;64:2352–60. doi: 10.2337/db15-0146 26050667 10.2337/db15-0146

