# Supplementary figures and images for: Brown adipose tissue plays a central role in systemic inflammation-induced sleep responses
Source: PLoS One. 2018 May 10;13(5):e0197409. doi: 10.1371/journal.pone.0197409 (PMC5945014; doi:10.1371/journal.pone.0197409)

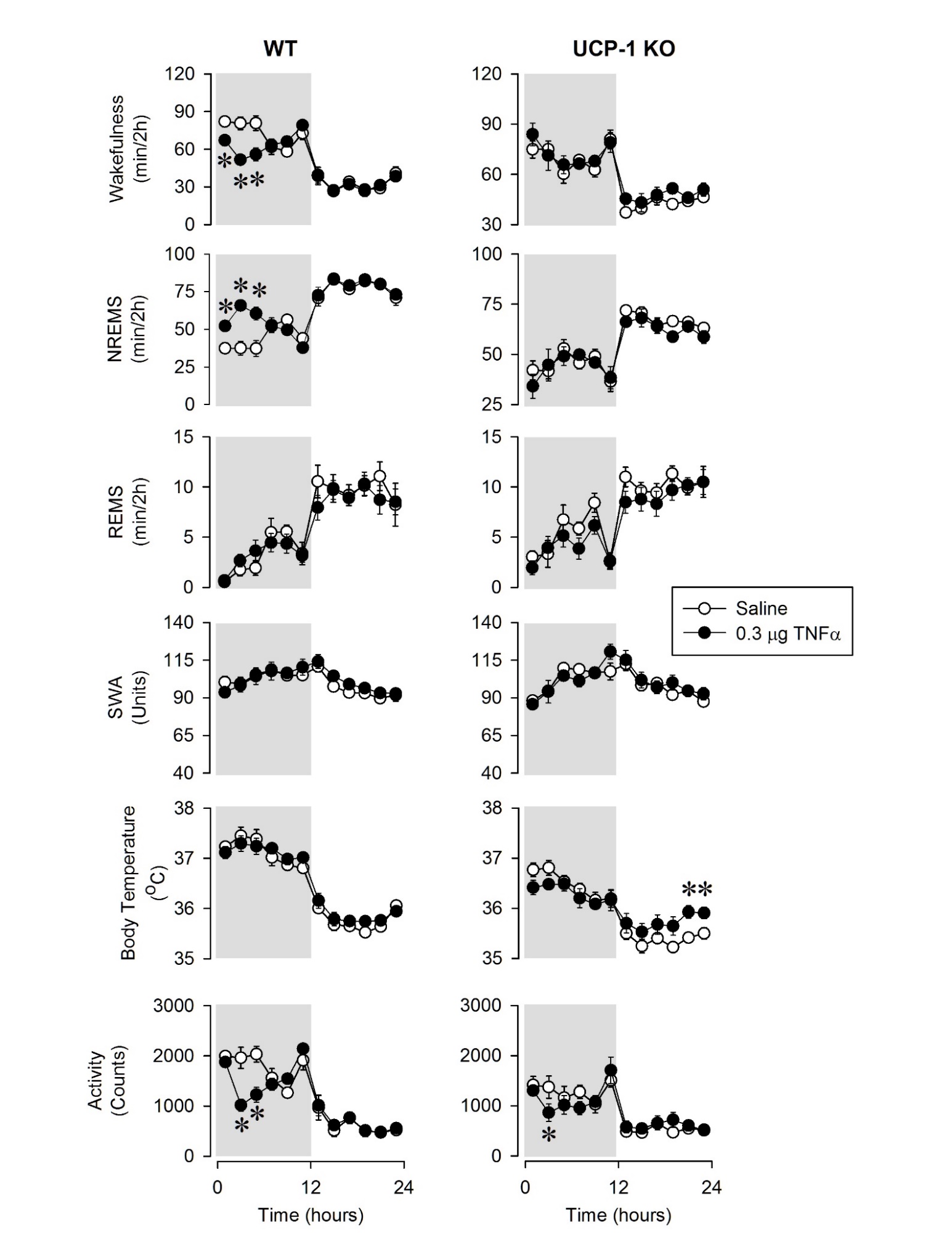


S1 Fig

Supplement: S1 Fig — Data are averaged in 2-h blocks. Shaded area: dark phase of the day; * significant difference from baseline (SNK test); error bars: SE. (DOCX) [file pone.0197409.s001.docx]

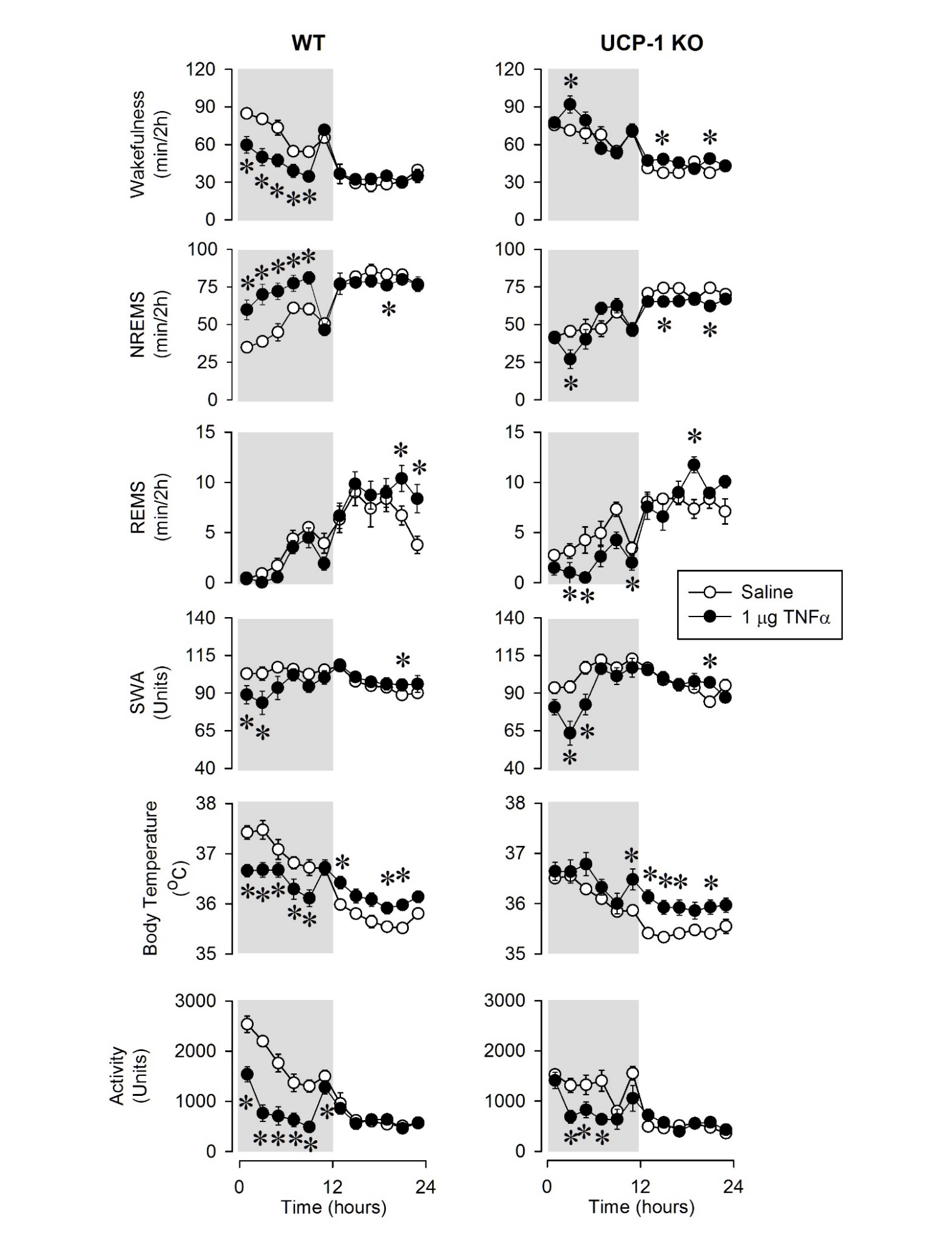


S2 Fig

Supplement: S2 Fig — See legends to S1 Fig for details. (DOCX) [file pone.0197409.s002.docx]

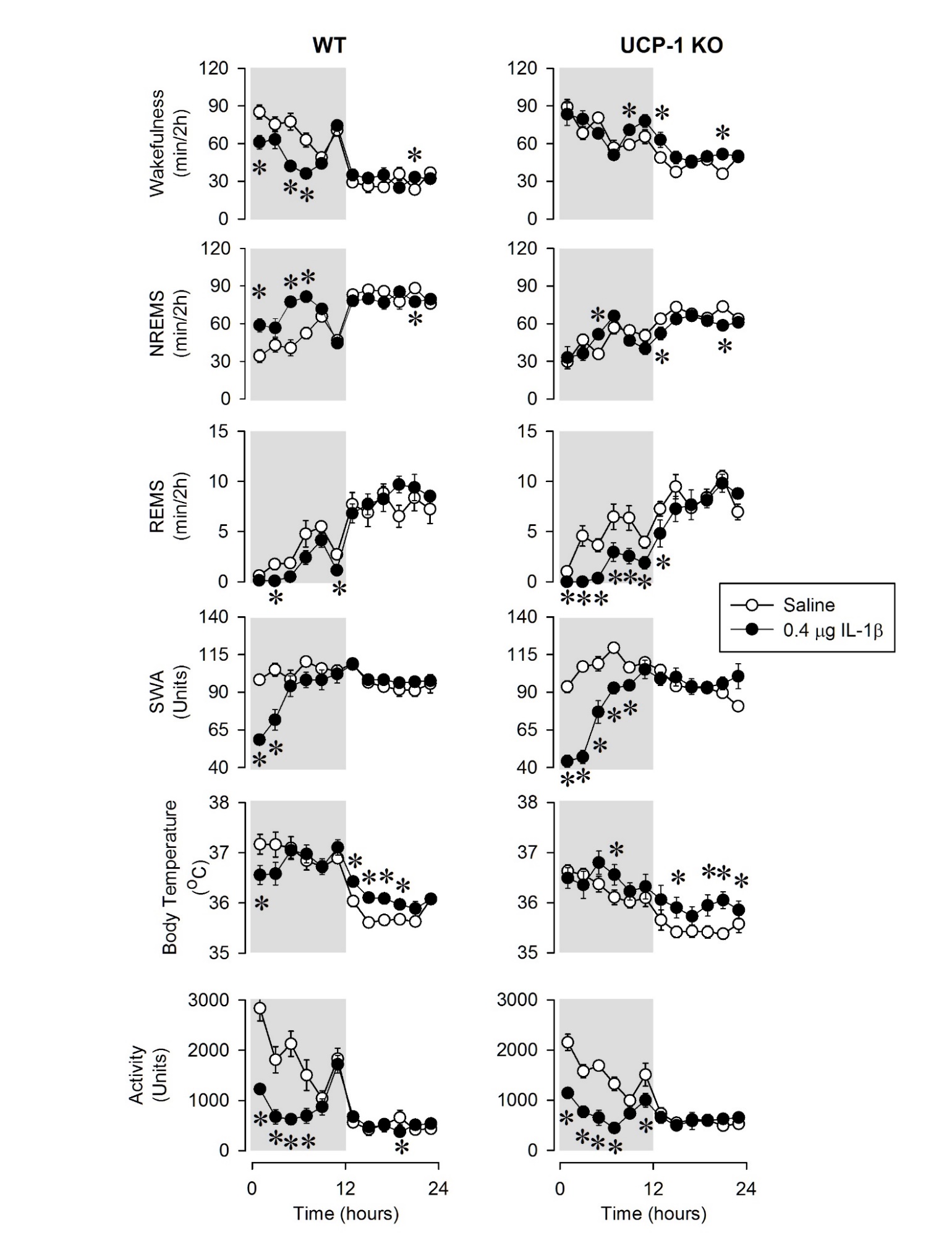


S3 Fig

Supplement: S3 Fig — See legends to S1 Fig for details. (DOCX) [file pone.0197409.s003.docx]

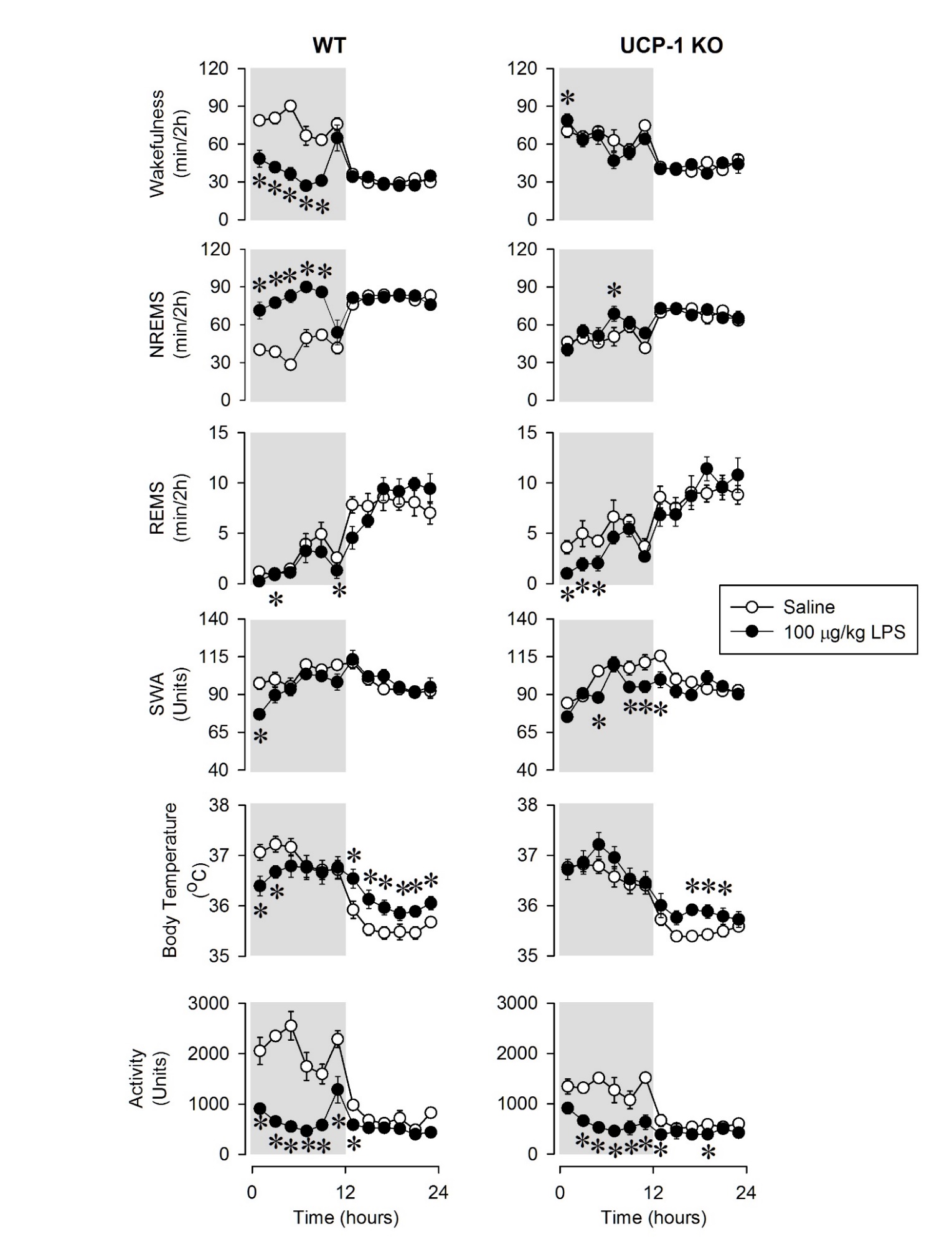


S4 Fig

Supplement: S4 Fig — See legends to S1 Fig for details. (DOCX) [file pone.0197409.s004.docx]

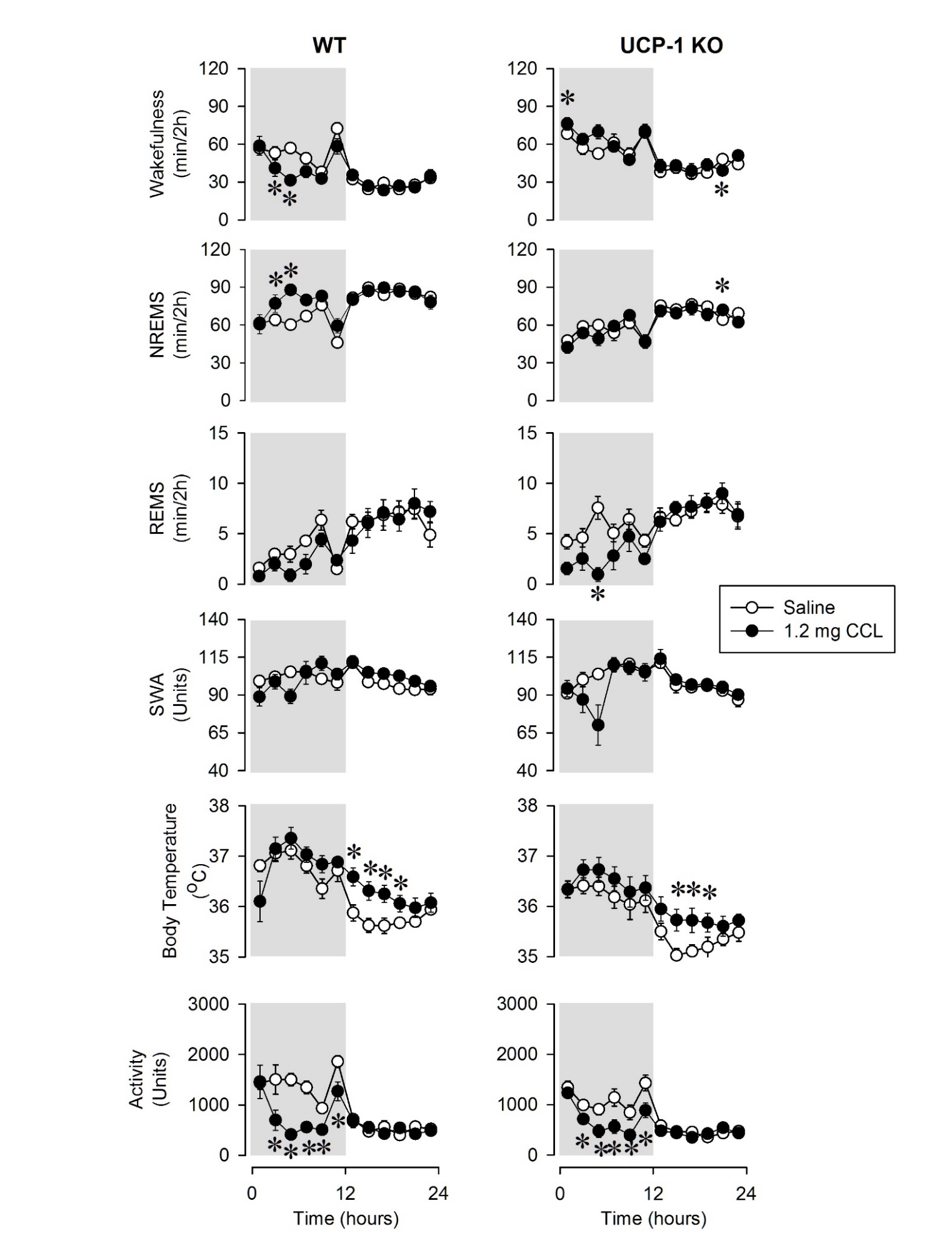


S5 Fig

Supplement: S5 Fig — See legends to S1 Fig for details. (DOCX) [file pone.0197409.s005.docx]

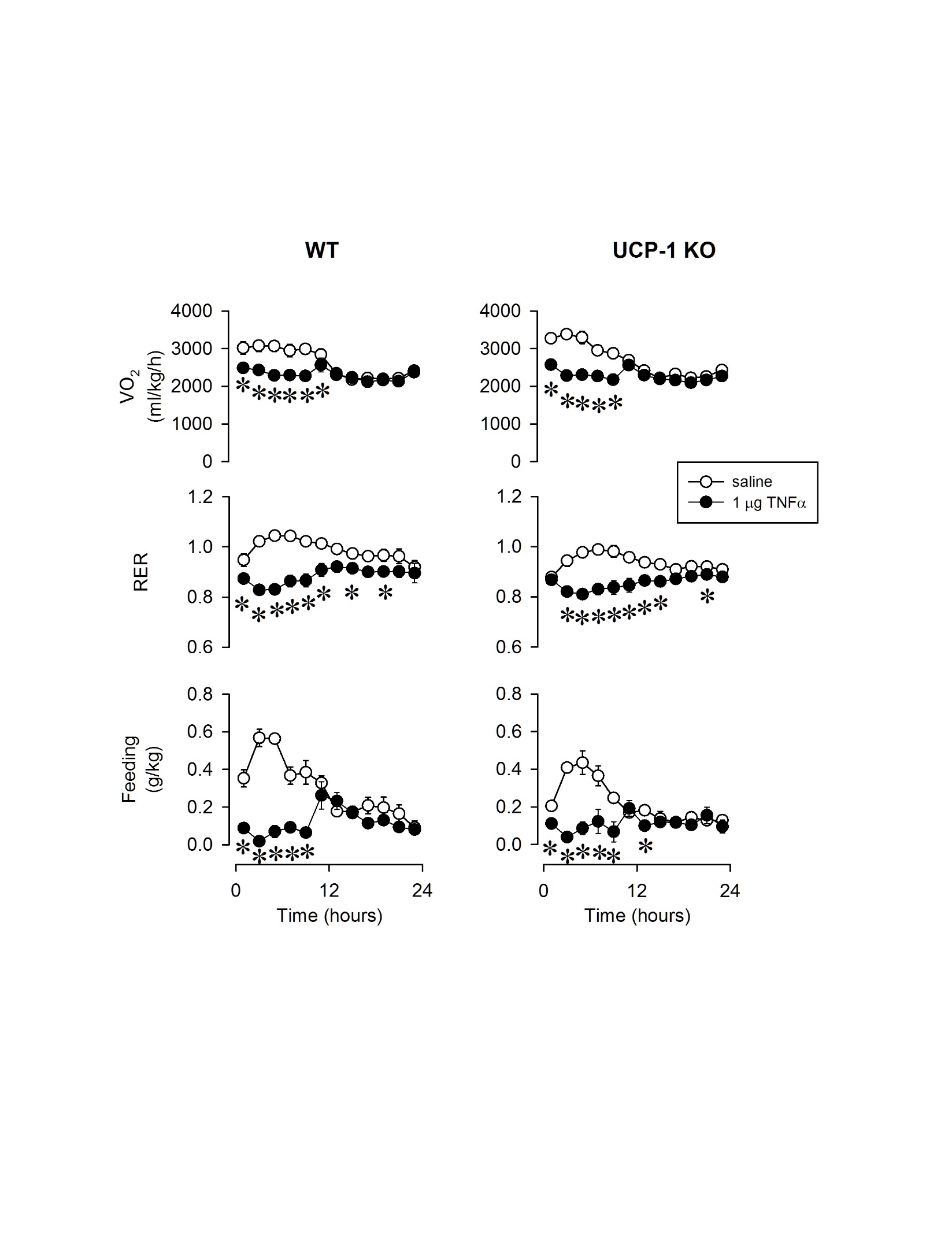


S6 Fig

Supplement: S6 Fig — See legends to S1 Fig for details. (DOCX) [file pone.0197409.s006.docx]
